# Rational Design of High-Number dsDNA Fragments Based on Thermodynamics for the Construction of Full-Length Genes in a Single Reaction

**DOI:** 10.1371/journal.pone.0145682

**Published:** 2015-12-30

**Authors:** Bhagyashree S. Birla, Hui-Hsien Chou

**Affiliations:** 1 Department of Genetics, Development and Cell Biology, Iowa State University, Ames, Iowa, United States of America; 2 Department of Computer Science, Iowa State University, Ames, Iowa, United States of America; Imperial College London, UNITED KINGDOM

## Abstract

Gene synthesis is frequently used in modern molecular biology research either to create novel genes or to obtain natural genes when the synthesis approach is more flexible and reliable than cloning. DNA chemical synthesis has limits on both its length and yield, thus full-length genes have to be hierarchically constructed from synthesized DNA fragments. Gibson Assembly and its derivatives are the simplest methods to assemble multiple double-stranded DNA fragments. Currently, up to 12 dsDNA fragments can be assembled at once with Gibson Assembly according to its vendor. In practice, the number of dsDNA fragments that can be assembled in a single reaction are much lower. We have developed a rational design method for gene construction that allows high-number dsDNA fragments to be assembled into full-length genes in a single reaction. Using this new design method and a modified version of the Gibson Assembly protocol, we have assembled 3 different genes from up to 45 dsDNA fragments at once. Our design method uses the thermodynamic analysis software Picky that identifies all unique junctions in a gene where consecutive DNA fragments are specifically made to connect to each other. Our novel method is generally applicable to most gene sequences, and can improve both the efficiency and cost of gene assembly.

## Introduction

**Fig 1:**
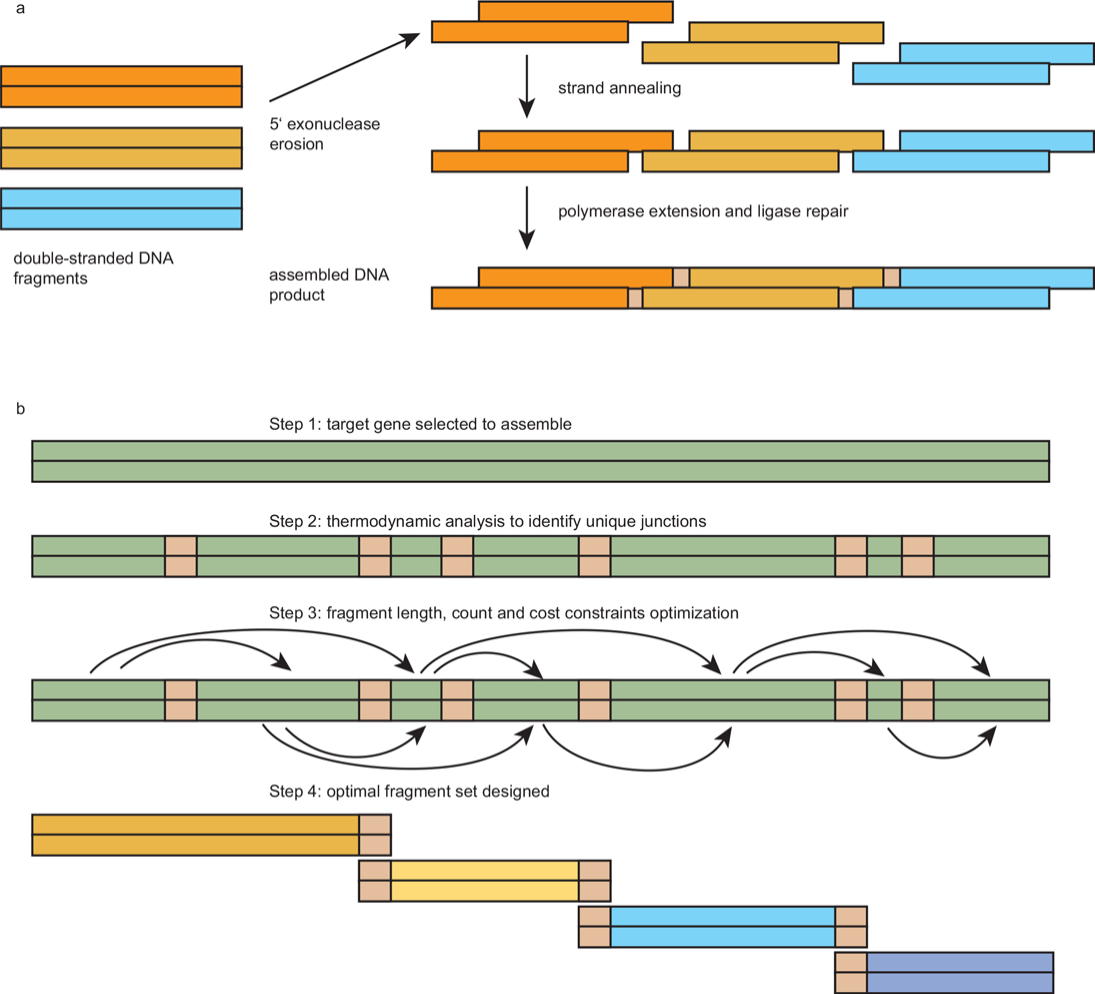
The Gibson Assembly method and Picky thermodynamic junction analysis. (a) The Gibson Assembly reagent includes three enzymes. The 5’ exonuclease erodes the 5’ ends on each dsDNA fragment, exposing single-stranded 3’ overhangs. The overhangs anneal to each other according to their compatible base-pairing. The DNA polymerase repairs gaps and the DNA ligase covalently binds the fragments to create a full-length product. (b) To design an optimal fragment set for gene assembly, the target gene is first analyzed using the Picky software to identify all its thermodynamically unique junction regions. Next, a separate Perl program takes these junction coordinates as well as some user specified design parameters such as acceptable minimum and maximum fragment lengths and the optimization goal for lower cost or fewer fragment count to finalize the optimal fragment set.

Synthetic biology is a new research field involving genes and genomes that are artificially designed, constructed and transformed into living cells [1]. The direct synthesis approach is preferable even for naturally occurring genes that can be cloned using recombinant DNA technologies, because it is often more efficient, reliable and flexible to synthesize genes rather than to clone them. Synthetic biology requires multiple hierarchical levels of assemblies starting from the smallest building blocks of short oligonucleotides to eventually reaching a full-length genome [2]. Several different gene assembly methods have been developed, including ligation-dependent assembly methods [3–6], the *FokI* method [7], variations of PCR-based methods [8–15], the BioBrick^TM^ assembly method [16,17], *in vivo* recombinant assembly methods [18,19] and the ligation cycling reaction method [20]. Among gene assembly methods, the relatively recent Gibson Assembly is one of the easiest ones to use [21,22], and has become a commercially available reagent kit from New England Biolabs (NEB Gibson Assembly Master Mix, #E2011). In the Gibson Assembly (see Fig 1A), three different DNA enzymes are optimally mixed together to assemble double-stranded (ds) DNA fragments: 1) a 5’ exonuclease, which shortens the 5’ end of DNA fragments and exposes a single-stranded 3’ overhang that can anneal to the other exposed DNA strands; 2) a DNA polymerase that fills in the missing DNA nucleotides after two strand annealing to repair the gaps; and 3) a DNA ligase that covalently repairs the nicks between two adjacent DNA fragments to make a single DNA molecule. Gibson Assembly has the following benefits: 1) the interior part of each DNA fragment is protected and cannot cause incorrect assembly because it remains double-stranded throughout the assembly process. This is in stark contrast to PCR based assembly methods where all strands in all DNA fragments are accessible to unintended hybridizations and may cause mis-assemblies during the repeated denaturing and re-annealing cycles; 2) Because all enzymes and DNA fragments required for assembly are mixed in at once, Gibson Assembly requires just a single step, a single tube, and about an hour reaction time; 3) It does not depend on specific DNA sequences (e.g., restriction enzyme recognition sites) for the assembly and it does not produce any *scar* in the resulted sequence; and 4) The assembled product can be used directly for many downstream steps, e.g., bacteria transformation (if a vector backbone is included in the DNA fragments assembled), restriction digestion for cloning, and PCR amplification.

Gibson Assembly is marketed to assemble larger dsDNA fragments that have been commercially synthesized from oligonucleotides. The vendor recommend against the assembly of more than 5 dsDNA fragments at once, and they report the maximum number of DNA fragments that have been assembled by this method is 12 (https://www.neb.com/faqs/1/01/01/how-many-fragments-of-dna-can-be-assembled-in-one-reaction). Although up to 52 single-stranded oligonucleotides can be assembled at once using Gibson Assembly [23], we have not found similar high-number dsDNA assemblies in the literature. Assembly of more DNA fragments commonly requires a hierarchical and multi-step approach by first assembling small subsets of DNA fragments and then combining the assembled longer DNA sequences to form even longer ones. We decided to investigate if Gibson Assembly kit and a recently derived kit called the NEBuilder HiFi DNA Assembly (NEB #E2621) have any inherent limitation on the number of dsDNA fragments that can be assembled at once. Our results prove that up to 45 dsDNA fragments can be assembled at once using these kits after we made some modifications to the standard protocols to limit the 5’ exonuclease activity on shorter dsDNA fragments.

Picky is a whole-genome thermodynamic analysis software commercially available but a version of it is free for the public [24,25]. It can efficiently compare all sequences of a large gene set and identify *thermodynamically unique* regions. The *uniqueness* is defined as having the highest difference between the melting temperature of a candidate probe when hybridizing to its target gene and the highest off-target melting temperature the same probe can establish with any other genes in the gene set. Note that probes achieving the highest melting temperature with their target genes may not necessarily prevent unintended off-target hybridizations if their melting temperatures to some other genes are also high, and Picky has the unique capability to estimate both the target melting temperature and the highest off-target melting temperature of each candidate probe. Picky design quality has been validated by its creator [26] and also by independent users [27,28]. In this study, we simply use Picky to identify all thermodynamically unique regions in a gene sequence to be assembled. Once all these regions are identified, DNA fragments for Gibson Assembly can be designed to connect only at these *junction regions*. The rationale is that the exonuclease exposed 3’ overhangs on each DNA fragment are similar to DNA probes that may potentially hybridize to other probes in the Gibson reaction buffer, but Picky analysis prevents them from hybridizing to unintended assembly partners. This idea is depicted in Fig 1B.

We have selected the green fluorescent protein, kanamycin resistance and tetracycline resistance genes to assemble in this study. We have chosen these genes because their correct assemblies may be easier to identify *in vivo* after being transformed into *E*. *coli*. This helps us quickly estimate the ratios of successful assemblies. Although it is possible to obtain oligonucleotides up to 200 base pairs (bps) nowadays, we have chosen to anneal the dsDNA fragments using shorter oligonucleotides averaging 50 bps. This increases the number of fragments to assemble without significantly increasing our cost. Since the dsDNA fragments are short, overlaps between them, i.e., the unique junctions, cannot be too long. For short fragment assemblies, the junctions represent significantly wasted resources. Each DNA fragment extends the assembly only by its length minus one of its junctions, e.g., a 50 bp fragment with 20 bp junctions extends only 30 bps on the assembled product. Economically, the junctions should be made as short as possible, but shorter junctions compromise the thermodynamic uniqueness of the junctions. We settled with 20 bp junctions in our experiments, but junctions of shorter or varied lengths should also work if they are analyzed by Picky. Table 1 shows more information about the selected genes, their lengths and number of fragments designed.

**Table 1:** Assembly dsDNA fragments designed for Green fluorescent protein gene (GFP), Kanamycin resistance gene (KanR) and Tetracycline resistance gene (TetR).

Gene names	Length (bp)	Unique junctions	Designed dsDNA fragments	Shortest fragment length (bp)	Longest fragment length (bp)	Average fragment length
**GFP gene**	755	27	27	40	60	48.03
**Kanamycin resistance gene**	953	36	28	42	70	53.32
**Tetracycline resistance gene**	1254	44	45	40	66	47.42

## Materials and Methods

### dsDNA fragment design

Download the free Picky software (http://www.complex.iastate.edu/download/Picky/index.html). Load target genes into Picky and click on its Probe design button. A design parameter window will show up. Picky was originally developed for microarray design, so its parameters were named in that context. Here we are using Picky for DNA fragment design so the parameter settings below are relevant to gene assembly purposes. Set the maximum and minimum oligo sizes to 20 or a different value for the preferred fragment junction size, the number of probe candidates to 200, and the number of probes per gene to 100. The latter two parameters just need to be large enough to instruct Picky to find all probe candidates that qualify as junctions. Also, set minimum match length to 6, minimum trigger similarity to 66% and salt concentration to 500 mM. These parameters increase the sensitivity level of Picky on short sequences and match the Gibson Assembly buffer condition better. Leave the rest of the Picky parameters in their default values. After the computation, save Picky probe design to an output file. Picky will create two files ending in.picky and.report; the.picky file will be used for the next step.

From the same website or the S1 File, obtain the Perl program (breaking_up_sequences_adding_restriction_sites.pl) and run it on the.picky file obtained in the previous step. This program takes 5 parameters: 1) the optimization goal for fragment count or synthesis cost (the cost formula is built into the program and can be modified), 2) the minimum acceptable fragment length, 3) the maximum acceptable fragment length, 4) the.picky filename created from the previous step, and 5) the original gene sequences analyzed by Picky. This program either reports an optimal DNA fragment set for each target gene, or reports failure given the chosen parameters and the limited number of thermodynamically unique junctions on certain genes. Users can adjust some parameters, usually by allowing longer dsDNA fragments, to try to obtain a working set. Optimization for cost involves exhaustive search and may take a very long time for certain junction distributions, whereas optimization for fragment count can always be efficiently performed. In Table 1, the relevant information of the three chosen genes and the design of their DNA fragments after Picky analysis is given. The longest tetracycline resistance gene has 1254 bps and is assembled from 45 dsDNA fragments averaging only 47 bps. The complete collection of fragment sequences are provided in S1 File.

### Modified Gibson Assembly protocol

Pairs of oligonucleotides at stock 10 μM concentration are annealed in equal molar volume by heating to 95°C and gradually cooling down to room temperature. A master mixture is then prepared from all annealed dsDNA fragments. Depending on the total number of fragments for each assembly, certain amount of the master mixture is added to pure water to make 10 μL, to which another 10 μL of Gibson Assembly or NEBuilder HiFi DNA Assembly master mix is added. The assembly reaction buffer is heated to 60°C for 4–8 minutes (min) and then cooled to 50°C for another hour in a thermal cycler. Following the assembly, a two-step PCR is used to amplify the assembled gene product (95°C for 2 min, followed by 30 cycles at 95°C for 15 sec and 68°C for 1 min/kb, and the final extension at 65°C for 5 min). The primers for polymerase chain reaction (PCR) are the forward oligonucleotide on the first fragment and the reverse oligonucleotide on the last fragment used in the assembly. The PCR amplicons are separated by agarose gel electrophoresis and the band at the expected assembly length, if visible, is purified and cloned into suitable vectors. The bands are purified using the QIAquick Gel Extraction Kit (Qiagen #28706). Both the non-specific TOPO TA cloning vector (Life Technologies #450030) and the more specific pGEM®-3Zf(–) vector that requires restriction-digestion cloning (Promega #P2661) have been successfully used to capture the assembled product. The assembled GFP gene contains the EcoRI and BamHI sites, and the assembled kanamycin and tetracycline resistance genes contain the HindIII and EcoRI sites. The restriction sites can be easily changed in the Perl program given earlier to avoid conflicting inner digestion sites on certain genes.

The pGEM®-3Zf(–) vector can be induced to express the assembled gene within an appropriate *E*. *coli* host via its *lacZ* promoter, thus allowing an efficient way to screen the antibiotic resistance genes. 1 μl of the plasmid with insert is transformed into NEB 5-alpha Competent *E*. *coli* (NEB #C2987) using the manufacturer recommended protocol. The transformed bacteria are spread on LB Ampicillin/X-gal/IPTG plates. The antibiotic ampicillin selects for the bacteria transformed with the plasmids. The blue-white screening using X-gal (5-bromo-4-chloro-3-indolyl-β-D-galactopyranoside) and IPTG (Isopropyl β-D-1-thiogalactopyranoside) helps select plasmids with assembled gene inserts. In the cases of kanamycin and tetracycline resistance gene assemblies, the corresponding antibiotics are also added onto the plates. After overnight incubation at 37˚C, colonies are picked and grown in liquid media. The plasmids are purified using the Qiagen QIAPrep Spin Miniprep kit (Qiagen #27106) and subsequently sequenced.

### 5’ exonuclease erosion test

10 μL of a 737 bp dsDNA fragment with initial concentration of 11 ng/μL is mixed with another 10 μL Gibson Assembly reagent master mix according to the manufacturer protocol. After 2, 4, 8 and 16 min at 50, 55 and 60°C, the reaction is immediately stopped by heating to 70°C for 20 min. Mung bean nuclease (NEB #M0250S) is then added according to manufacturer instructions to remove 3’ DNA overhangs. Subsequently the mung bean nuclease is inactivated by adding in 1% SDS. The sample is purified to remove all enzymes and buffers and 1 μL of each sample containing the eroded dsDNA fragments is run through the BioRad Experion™ Automated Electrophoresis System with gel-on-a-chip technique to precisely determine their lengths. Each combination of timing and temperature is repeated a few times to average the values.

## Results

### Modifications to standard Gibson Assembly protocol

The standard protocol for Gibson Assembly recommended by the manufacturer involves a simple mixing step of the assembly reagent buffer with all dsDNA fragments and an isothermal reaction time of up to 1 hour at 50°C. If a digested plasmid backbone is included in the fragments, the assembled product can be directly transformed into *E*. *coli*. However, this standard protocol did not work for the assembly of high-number dsDNA fragments that we have tested. We considered all possible causes of failures and hypothesized that the following two were the most likely problems: 1) the 5’ exonuclease in the assembly reagent buffer eroded too many nucleotides, thus rendering the fragments single-stranded that cannot be precisely connected to each other; and 2) the yield of the assembled product was low due to the high-number of DNA fragments that must come together and the more diluted concentration of each fragment in the assembly.

The exact erosion speed of the 5’ exonuclease in the Gibson Assembly reagent buffer is not known. The manufacturer recommends that overlaps between dsDNA fragments to be 100 bps or less, suggesting that up to 100 bps can be removed by the 5’ exonuclease under the standard protocol. Because our DNA fragments are much shorter than 100 bps, they can all be reduced to single-stranded DNAs by the exonuclease. To limit the exonuclease erosion to the 20 bp designed junctions between shorter DNA fragments, we conducted an assay with varied temperature and reaction time combinations as described earlier to identify the optimal erosion condition. The results are given in Table 2. Up to 3 replicates were performed for each combination of temperature and reaction time. Surprisingly, the different temperatures and reaction times do not seem to produce significantly different erosion lengths. Reaction time seems to play a role only at 50°C; at 55 and 60°C they seem to have much lesser influence on erosion lengths. At higher temperatures, the 5' exonuclease seems to degrade very quickly; thus prolonged exposure at higher temperatures no longer produces shorter fragments. However, at higher temperatures the exonuclease also seems more active, thus eroded fragments are shorter at 55 and 60°C than at 50°C under the same reaction time. Finally, at 50°C, the 5'-exonuclease seems to have longer life, thus the fragments continue to be eroded, which supports our first hypothesis.

**Table 2:** Gibson Assembly 5’ exonuclease erosion length under different temperature and reaction time.

Temperature	Reaction time in minutes to erode a 733 bp DNA fragment			
	2	4	8	16
50°C	Avg. length 691	Avg. length 696	Avg. length 685	Avg. length 682
	Std. Dev. 16.26	Std. Dev. 14.15	Std. Dev. 10.41	Std. Dev. 11.15
	Erosion 21.3	Erosion 18.3	Erosion 24.2	Erosion 25.7
55°C	Avg. length 678	Avg. length 677	Avg. length 675	Avg. length 672
	Std. Dev. 8.74	Std. Dev. 7.21	Std. Dev. 7.02	Std. Dev. 5.13
	Erosion 27.7	Erosion 28	Erosion 28.8	Erosion 30.7
60°C	Avg. length 674	Avg. length 672	Avg. length 671	Avg. length 667
	Std. Dev. 6.03	Std. Dev. 4.51	Std. Dev. 5.20	Std. Dev. N/A
	Erosion 29.3	Erosion 30.7	Erosion 31.0	Erosion 33.0

The required 3’ overhang is 20 bps on both ends of a DNA fragment in our assembly, but this is the lower bound. Given the unevenness and randomness in 5’ exonuclease erosion as observed in Table 2, a slightly longer erosion length is preferred. Because erosion at 60°C seems to have the best control over erosion length with the smallest standard deviations, we chose to run all subsequent assembly reactions for 4 or 8 min at 60°C before reducing the reaction temperature to the standard 50°C for an hour.

Successful high-number Gibson Assembly critically depends on the final fragment concentration at assembly. If it is too high the 5’ exonuclease can be exhausted before all 3’ overhangs are exposed, thus preventing a successful assembly. If it is too low the yield of the full-length assembly product will become extremely low and may fail to PCR amplify efficiently. Gibson manufacturers recommend the final fragment concentration of 200–1000 nanomolar (nM) for assembly of 4–6 fragments. This value will likely vary if the number of fragments are increased. We tried a range of fragments concentrations and tabulated the ones that worked for all three genes. Our successful high-number assemblies all have final fragment concentrations in the low 3–8 nM range which are depicted in Table 3. Even with optimal fragment concentrations the assembly products are too low to be harvested. To resolve the low assembly product yield problem, we added a PCR step after the assembly.

**Table 3:** Final dsDNA fragment concentration (conc) in successful assemblies.

Gene	Number of fragments	Volume of each 10 μM ssDNA added to anneal (μL)	Mastermix used in assembly (μL)	Water added (μL)	Conc of each fragment after annealing (μM)	Conc of each fragment in master mix (μM)	Final conc of fragment in Gibson Assembly (μM)
**GFP**	27	9	1	9	4.5	0.167	0.008
**KanR**	28	1	9	1	0.5	0.018	0.008
**TetR**	45	2	3	7	1	0.022	0.003

### Assembly outcomes and validations

**Fig 2:**
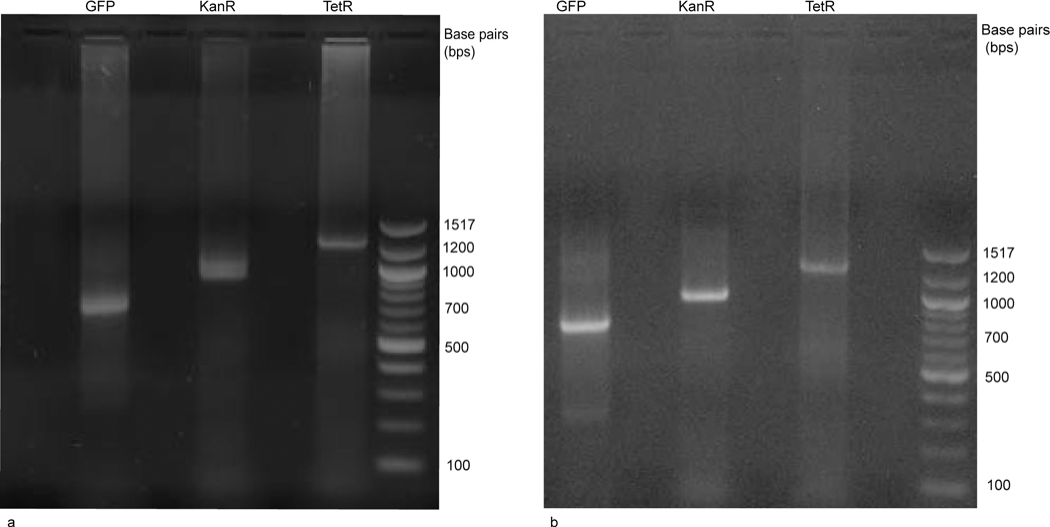
Agarose gel electrophoresis of the three assembled genes (a) The first lane contains the GFP gene assembled from 27 dsDNA fragments showing up at the expected 757 bp length. The second lane contains the kanamycin resistance gene assembled from 28 dsDNA fragments showing up at the expected 953 bp length. The third lane contains the tetracycline resistance gene assembled from 45 dsDNA fragments at the expected 1254 bp length. All assemblies were performed using Gibson Assembly master mix. (b) The same assemblies performed using the NEBuilder HiFi DNA Assembly master mix. The agarose gel is stained with ethidium bromide.

Initial attempts to assemble the GFP gene failed until we started using the modified Gibson Assembly protocol. Subsequently the kanamycin and tetracycline resistance genes were also assembled using this protocol. The successful assemblies can be visualized in agarose gel as shown in Fig 2. Subsequently, the gel bands were purified, cloned into plasmid vectors and transformed into *E*. *coli*. The assembly results were then confirmed by sequencing. This assembly process has been repeated a few times for all three genes and most repeats produced a few 100% correctly assembled sequences confirmed by sequencing.

To assess the quality of high-number Gibson Assembly, we further conducted the following experiments. Our original plan for the GFP gene was to count *E*. *coli* colonies that fluoresce after the induction of GFP expression from the inserted plasmids. However, the fluorescence signal in bacteria colonies was too weak to be picked up by the imaging system. Alternatively, we randomly picked 83 colonies for sequencing and found 33 of them (about 40%) carry the correct GFP gene sequence. The success rate of the kanamycin resistance gene assembly was measured by plating equal amount of the transformed *E*. *coli* cell culture both on Petri dishes that contain only the antibiotic ampicillin and Petri dishes that contain both ampicillin and reduced concentration of the antibiotic kanamycin. Ampicillin selects bacteria that have acquired the inserted plasmid, and kanamycin selects bacteria that contain the correctly assembled kanamycin resistance gene when the bacteria were induced to express the kanamycin resistance gene. The tetracycline resistance gene was validated in a similar fashion. The validation results and the success rate estimates for high-number Gibson Assembly is summarized in Table 4.

**Table 4:** Assembly quality assessment for the three assemblies.

Assembled gene	Validation method	Colony counts	Sequencing confirmation	Perfect assemblies
**Assembled using the Gibson Assembly Kit**				
GFP gene	83 sequencing runs	N/A	33 out of 83 are confirmed correct	39.76%
Kanamycin resistance gene	Spread on 3 Amp plates and 3 Amp/Kan plates	6 colonies on the Amp/Kan plates compared to 56 colonies on the Amp only plates	1 colony–perfect sequence, 5 colonies– 1 base error	1.7%
Tetracycline resistance gene	Spread on 3 Amp plates and 3 Amp/Tet plates	2 colonies on the Amp/Tet plates compared to 21 colonies on the Amp only plates	Various base pair errors	0%
**Assembled using NEBuilder HiFi Assembly Kit**				
Kanamycin resistance gene	Spread on 3 Amp plates and 3 Amp/Kan plates	4 colonies on the Amp/Kan plates compared to 15 colonies on the Amp only plates	2 colonies–perfect sequence, 2 colonies– 1 base error	13.3%
Tetracycline resistance gene	Spread on 3 Amp plates and 3 Amp/Tet plates	3 colonies on the Amp/Tet plates compared to 15 colonies on the Amp plates	All 3 colonies have perfect sequence	20%

Working concentrations: Ampicillin 100 μg/mL, Kanamycin 50 μg/mL, Tetracycline 6.25 μg/mL

The concentration of kanamycin and tetracycline were reduced on the selection plates because at their full strength we never obtained any colony. In order to understand the assembly errors, we reduced the antibiotic concentrations to obtain some colonies that we can sequence and assess their errors. Using the Gibson Assembly master mix, we found it harder to assemble the tetracycline resistance gene from 45 dsDNA fragments. We repeated this assembly a few times but have never obtained perfect assembly.

New England Biolabs released a new reagent kit (NEBuilder HiFi DNA Assembly Master Mix, # E2621L) while we were performing the Gibson Assembly studies, and they claimed that this improved assembly reagent mix has higher accuracy and efficiency than the Gibson Assembly Master Mix. We decided to test this new reagent kit on the same fragment sets of the kanamycin and tetracycline resistance genes to check if it would improve our results. GFP gene was not constructed again using this kit as it has already had a decent percentage of correct assemblies using the Gibson Assembly kit. The perfect assemblies using the NEBuilder HiFi DNA Assembly Mix are also summarized in Table 4. Using this master mix, the tetracycline resistance gene was repeatedly assembled with no errors. Because the NEBuilder HiFi DNA Assembly Master Mix is compatible with Gibson Assembly protocols, our thermodynamic design method is equally applicable to both kits.

To assess if there is any statistical significance between the different number of perfect assemblies among genes, the two-tailed Fisher exact test was performed and yielded a p-value of 0.6648 between kanamycin resistance gene and tetracycline resistance gene when using NEBuilder HiFi DNA kit and a p-value <0.0001 between GFP and kanamycin resistance gene when using the Gibson Assembly kit. Therefore, we do not have evidence that the number of perfect assemblies vary with respect to any particular gene assembly using the NEBuilder HiFi DNA Assembly kit, but the results are significantly different for the Gibson Assembly kit at 5% significance level between the two genes tested. Thus, our method can potentially be useful for the construction of other genes using the NEBuilder HiFi Assembly kit.

## Discussion

The previously reported maximum Gibson Assembly included 52 oligonucleotides in a single-stranded assembly of a viral gene, but the authors specifically advised against using their fragment design method for any other purpose [23]. The manufacturer of the Gibson Assembly reagent kit actually advises against using more than 5 fragments in an assembly. Furthermore, Gibson Assembly is more commonly used to assemble from longer dsDNA fragments that are more than a couple hundred bps and have been *bootstrap-assembled* from basic oligonucleotide building blocks by commercial vendors. When assembling from longer dsDNA fragments, Gibson Assembly works very well as it can tolerate more variable 5’ exonuclease erosions and less than optimal but longer fragment junctions.

We have developed a general purpose fragment design method that is applicable to any gene construction, and in our tests, up to 45 dsDNA fragments can be assembled at once. For assemblies of the GFP and kanamycin resistance genes using less than 30 fragments, our results are reproducible and with sufficient yields of perfectly assembled sequences after PCR. Using the NEBuilder HiFi Assembly, we also have perfectly assembled sequences of the tetracycline resistance gene from 45 fragments. Although different fragment sets likely have distinct difficulties to assemble even with the same number of fragments, we learned from our study that somewhere between 30 to 45 fragments lies the practical limit of Gibson Assembly—Beyond that point the success rate will drop significantly and it is no longer feasible to attempt higher-number assemblies.

**Fig 3:**
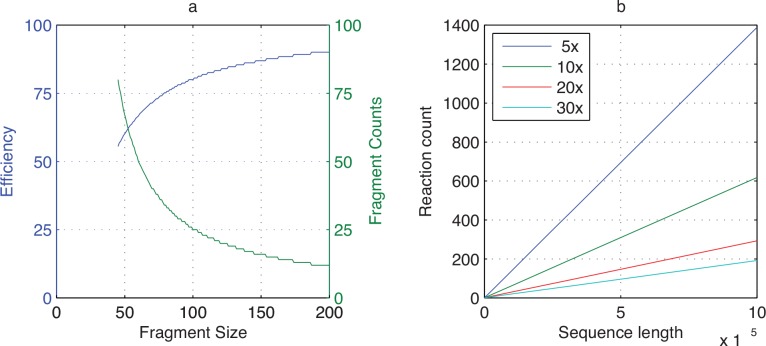
Assembly efficiency and reaction count under different conditions. (a) The assembly efficiency and fragment count to assemble a 2000 bp gene using different fragment sizes. (b) The assembly reactions required to assemble sequences up to a million bps from 200 bp fragments under different Gibson Assembly capacities up to 30 fragments at once. In both figures the junction length between fragments is fixed at 20 bps and it is assumed that any sequence to assemble can be evenly divided by the fragments.

In this study, our main focus is to test if we can significantly increase the number of dsDNA fragments that can be assembled at once. The reason we chose to create shorter dsDNA fragments from complementary oligonucleotides is to avoid increasing the length of the assembled gene products—it will cost a lot more to synthesize longer dsDNA fragments and the assembled sequences will be harder to validate if they must go through shotgun assembly. We must point out that this is our strategy to test high-number assemblies and is not a very efficient way to directly assemble genes because a significant fraction of junction regions were synthesized twice and wasted. In practice, the longer the individual fragments, the more efficient the assembled sequences can be elongated. Assuming 20 bp junctions between all fragments and a 2000 bp gene can be evenly divided among fragments of any sizes, as seen in Fig 3, it requires 80 fragments of 45 bps to assemble the gene with an efficiency less than 56%, but it only requires 12 fragments of 200 bps to assemble the same gene with an efficiency above 90%. Because the interior of each DNA fragment is protected by double-strand and does not interfere with the assembly process, in principle the length of each fragment should not significantly increase the assembly difficulty. Therefore, longer genes can be assembled using longer fragments when designed with our method. Further studies will have to be conducted to test high-number gene assemblies using longer fragments.

**Fig 4:**
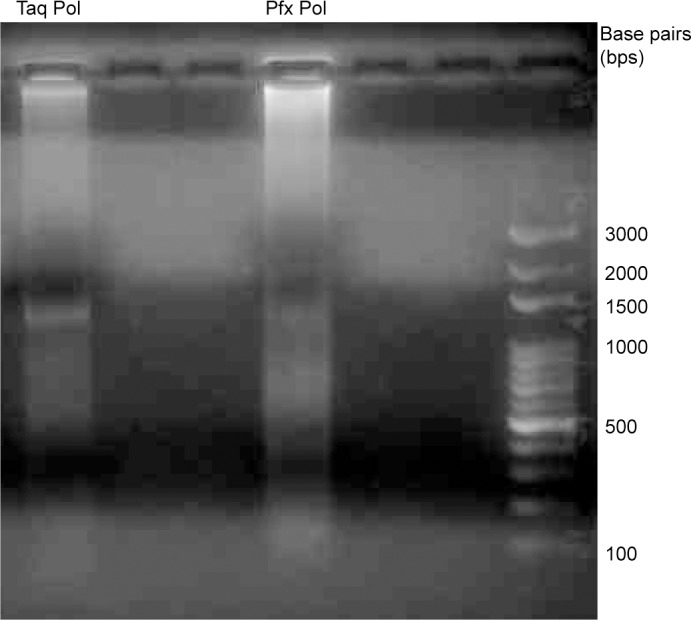
Difference in DNA polymerase behaviors. The tetracycline resistance gene assembly product was PCR amplified by Taq DNA polymerase (Lane 1) and Pfx DNA polymerase (Lane 2). Pfx amplification caused polymerization and produced some high molecular weight products, thus the Taq polymerase was chosen for subsequent studies. The last lane contained the 100-bp DNA ladder.

We also tried to discern if there are any assembly error patterns. Most of the errors in the assembled genes were single-base mutations or deletions. Originally, we thought some errors could occur in the fragment junctions because these are where the assembly activities happened. However, we found errors can occur anywhere in the fragments, including those interior regions protected by double-strand. Some of these errors could have been present in the original oligonucleotides that were used for assembly because it is known that oligonucleotide synthesis can be imperfect [29]. We also suspect that some of the errors might have been introduced during the PCR amplification of assembled products. We have used both the Taq DNA polymerase (NEB #M0273) and Pfx high fidelity DNA polymerase (Life Technologies #11708) for the PCRs, and we have found Pfx can produce polymerization products with high molecular weight (Fig 4), so we mainly used Taq in most of our studies. Since Taq has no 3’ exonuclease proof-reading activity, that may have increased the errors due to lingering unassembled fragments in the buffer. If the PCR amplification step can be avoided, it is reasonable to expect that fewer assembly errors will occur. We are going to study other assembly methods that might allow higher fragment concentration to begin with, thus the assembled products may not require PCR amplification. If successful, this will improve the assembly quality.

One of the Gibson Assembly papers demonstrated the assembly of a 582,970 bps *Mycoplasma genitalium* genome from about 10,000 oligonucleotides averaging 50 bps in length [2]. The assembly started with 101 cassettes that had been assembled by commercial vendors and were approximately 5000–7000 bps in length. It took 5 hierarchical assembly steps and 40 assembly reactions, each including up to 5 fragments, to produce the full-length genome from the cassettes—If 28 fragments could be assembled at once, it would only require 4 reactions to complete the assembly. Assuming a one million bp genome can be assembled from evenly distributed 200 bp fragments with 20 bp junctions between them, if only 5 fragments can be assembled at once, the whole 5556 fragments will take 1389 reactions to assemble, but if 30 fragments can be assembled at once, it will only take 192 reactions to obtain the one million bp genome (Fig 3B). In this study, we demonstrated that using thermodynamic analysis to discover unique junctions between each consecutive DNA fragment will allow high-number fragment assemblies. Since the Picky thermodynamic analysis is not specific to Gibson Assembly chemistries, the same method should be equally applicable to other assembly methods where unique pairwise DNA strand hybridizations are required. With our techniques, much longer genes can be synthesized with more fragments in one step. This may improve both the cost and quality of gene synthesis.

## Supporting Information

S1 File The Perl code to process.picky output file licensed under GPL 2.0, the three gene sequences and their respective sets of designed dsDNA fragments are included. (ZIP)
